# Revisiting Methicillin-Resistant *Staphylococcus aureus* Infections

**DOI:** 10.4103/0974-777X.59251

**Published:** 2010

**Authors:** Abdelkarim Waness

**Affiliations:** *Division of Internal Medicine, King Abdulaziz Medical City, Department of Medicine, Code #1443, P.O. Box 22490, Riyadh 11465, Saudi Arabia*

**Keywords:** Blood cultures, linezolid, methicillin-resistant *Staphylococcus aureus*, sepsis, vancomycin

## Abstract

Within less than 50 years, methicillin-resistant *Staphylococcus aureus* (MRSA) made a tremendous impact worldwide. It is not limited to medical facilities and healthcare institutions anymore. Indeed since two decades, cases of MRSA infections arising from the community among apparently healthy individuals are increasing. In this paper, I will present a case of community-associated MRSA sepsis followed by a comprehensive review about the history, pathogenesis, epidemiology, clinical presentations, diagnostic modalities, therapeutic options, contributing factors, growing cost and other pertinent elements of this newly evolving epidemic of MRSA infections.

## INTRODUCTION

Methicillin-resistant *Staphylococcus aureus* (MRSA) infections are gaining more attention lately. Traditionally confined to medical institutions, a growing number of community-acquired cases are being diagnosed. Among the different forms of pathologic presentations, MRSA sepsis and pneumonia can be lethal. Only few costly antibiotics are currently efficient in treating these infections. It remains unclear, however, whether MRSA will expand the scope of their resistance thus causing further severe infections and posing growing challenges to the medical community.

## CASE PRESENTATION

A 70-year-old man, known only to have dyslipidemia, was admitted to our hospital because of fever associated with fatigue and nausea for 6 h. His medications were atorvastatin and omeprazole. He had no prior history of intravenous drug abuse. On examination, the patient was slightly drowsy but in no acute distress. His vital signs were: temperature 38.7°C, pulse 94 bpm, respiratory rate 20 per min, BP 123/59 mmHg and oxygen saturation 100% on room air. His overall exam was unremarkable. The patient did not have skin rash, signs of meningismus or a cardiac murmur. His laboratory findings were: WBC 7.0×10^6^/L, hemoglobin 132 g/L, platelets 125×10^6^/L, random glucose 16.9 mmol/L, sodium 132 mmol/L, potassium 3.6 mmol/L, bicarbonate 20 mmol/L, serum creatinine 88 micromol/L, ALT 69 U/L, AST 87 U/L and GTP 155 U/L. The rest of his laboratory work-up, including urinalysis, coagulation profile and hepatitis profile, were unremarkable. Chest X-ray was normal. Transthoracic echocardiogram did not show vegetations or valvular lesions. The patient was started on intravenous ceftriaxone and vancomycin. Two days later, two of his blood cultures grew MRSA. His intravenous antibiotic regimen was changed to linezolid and cefipime. The patient finished 2 weeks of this regimen with obvious clinical and laboratory improvement and was discharged home in good condition.

## HISTORY

**Figure 1:**
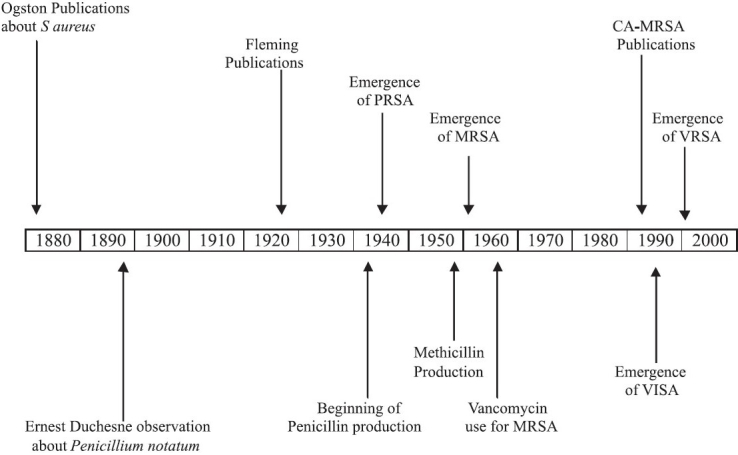
Evolution chronology of methicillin-resistant *Staphylococcus aureus*

It is likely that throughout history the bacterium *Staphylococcus aureus* (*S. aureus*) has accompanied mankind. It causes multiple well-known diseases to human societies, such as impetigo, boils and abscesses. The first published observations about *S. aureus* infections were performed by Ogston between 1880 and 1882.[1] Treatment for these infections was limited until 1896 when, a French medical student, Ernest Duchesne, observed that colonies of *S. aureus* could be destroyed by the mold *Penicillium notatum*. This observation was rediscovered by bacteriologist Alexander Fleming in 1928. Dr. Fleming published the results of his investigations in 1929, noting that his discovery might have therapeutic value if it could be produced in quantity. Ten years later, limited penicillin extraction was started at Oxford University. In 1943, a full large-scale production of the “miracle drug” began in the United States of America.[2] Four years later cases of Penicillin-resistant *Staphylococcus aureus* (PRSA) were observed. From that point on, the race to win the fight between this ever-evolving bacterium and scientists has been intense. In 1959, methicillin was discovered to counter the spread of PRSA. This victory against *S. aureus* was short-lived. Indeed, in 1961, the first cases of MRSA were reported in the United Kingdom; the “superbug” was just born.[3] Shortly thereafter, MRSA became pandemic in many medical institutions worldwide. It was dubbed hospital-associated MRSA (HA-MRSA) [Figure 1].

Vancomycin (derived from the word “vanquished”) was developed from soil samples obtained from the jungles of the island of Borneo in the 1950s. It was approved by the U.S. Food and Drug Administration in 1958. It proved to be active against many bacteria. However, because of its side-effects and intravenous delivery, it was only used as a second-hand antibiotic. That changed by the late '60s with the spread of HA-MRSA. Vancomycin reemerged then as “the antibiotic of last resort.”[4] This glycopeptide antibiotic came to the rescue of many patients infected with MRSA for four decades. However, in the late '90s, Cases of vancomycin-intermediate *S.aureus* (VISA) were observed. This phenomenon worsened by the emergence of vancomycin-resistant *Staphylococcus aureus* (VRSA) later on.[5] This dangerous antibiotic-resistance development occurred close to another worrisome important milestone in the history of MRSA. Indeed, and in the early 90s, cases of community-associated MRSA infections (CA-MRSA) were reported in Western Australia among apparently healthy indigenous people.[6] Since then, more CA-MRSA infections are diagnosed worldwide.

## PATHOGENESIS

*S. aureus* belongs to the Staphylococci family. It is a Gram-positive coagulase-positive nonmotile spherical bacteria, 1 μm in diameter, which usually clumps in clusters (from the Greek, staphyle = bunch of grapes). It is a pyogenic pathogen that does not form spores and is facultatively anaerobic. It colonizes the nares and axillae. S. aureus expresses many potential virulence factors, including surface proteins that promote colonization of host tissues, factors that probably inhibit phagocytosis (capsule, immunoglobulin-binding protein A) and toxins that damage host tissues and cause disease symptoms.[7]

The classification of the MRSA strains is complex and is still evolving. Resistance to methicillin and other beta-lactam antibiotics is caused by the mecA gene, which is situated on a mobile genetic element, the Staphylococcal Cassette Chromosome mec (SCCmec). To date, seven SCCmec types (I–VII) have been distinguished, and several variants of these SCCmec types have been described. The early MRSA clones were HA-MRSA. However, from the late 1990s, CA-MRSA clones emerged worldwide. Initially, CA-MRSA was both phenotypically and genotypically different from HA-MRSA. CA-MRSA harbors SCCmec type IV, V or VII. However, more recently, the distinction between HA-MRSA and CA-MRSA has started to fade.[8] Panton-Valentine leukocidin is a cytotoxin found in MRSA. Its contribution to the virulence of the organism is still debated among scientists.[9]

## EPIDEMIOLOGY

Many health care providers still think about intensive care units and acute care settings when they hear the labeling MRSA. Nowadays, the epidemiology of MRSA is changing. Recent studies have demonstrated that MRSA is prevalent in livestock animals and slaughterhouses in many European countries, Canada and Singapore.[10–11] Not only that, samples taken from American beaches show the presence of thriving MRSA in sea water. The origin of these bacteria remains unknown because there are no swimmers in those places.[12]

Studies conducted in different parts of the world confirm the increase in MRSA infection prevalence. This worsening resistance trend is seen in countries with large health resources as well as countries with modest ones. In a 15-year longitudinal assessment conducted by Seal and associates at a large hospital in Chicago, the annual rate of resistance to methicillin was found to increase from 13% in 1986 to 28% in 2000 and has not subsided.[13] A similar observation was put forward by another study conducted in the small Caribbean Island nation of Trinidad, the increase rate being 12.5% in 1999 to 20.8% in 2004.[14] Another Mexican study showed a substantial increase from 37% to 49%.[15] This worrisome trend is compounded by another severe observation: transmission of MRSA to healthy individuals. A recent French study suggests a transmission rate of nearly 20% from hospital-discharged patients to household contacts.[16] Hence, MRSA carriers are also on the rise, causing further spread of this threatening organism.

### Mortality/morbidity

MRSA infections come in many shapes and forms. Therefore, morbidity and mortality rates are dependent on the type of infection and other factors such as patient's age or the presence of comorbidities. When compared with methicillin-sensitive *S. aureus*, MRSA infections, especially severe ones, are associated with significant increases in length of hospitalization and hospital charges.[17] This usually translates in higher in-hospital as well as after hospital discharge mortality rates.[18] The mortality rate is higher in individuals 65 years or older. In 2005, around 94,000 invasive MRSA infections were estimated to occur in the United States of America, causing about 18,500 deaths.[19]

### Gender/age

Invasive MRSA infections can be observed in all age spectrums. Neonates,[20] middle-aged individuals as well as the elderly can be affected.[21] However, the rate of rate of infection is highest among patients 65 years and older. These infections are more common in Blacks than in Whites, with a male predilection.[22–23]

## PREDISPOSING FACTORS

Since its emergence in the early sixties, MRSA was recognized to be associated with some predisposing factors. Hospitals and health care facilities became a breeding ground for this resistant pathogen. They not only form an immense reservoir but also provide a fertile ground for its transmission by health care workers.[24] MRSA seems to thrive in places with unsanitary conditions. Nursing homes, with their growing elderly residents, are ideal for its growth.[25–26] Places with overcrowding, such as prisons and dormitories, can facilitate the spread of MRSA.[27] Farm workers, especially those working with pigs, have apparently higher MRSA exposure.[28] Other predisposing factors for different types of invasive infections by this “superbug” include individuals infected with human immunodeficiency virus,[29] men having sex with men,[30] intravenous drug users[31] and homeless people.[32]

The occurrence of CA-MRSA since the early nineties has changed the spectrum of MRSA infections. Indeed, more cases of these severe infections are being reported in apparently healthy individuals with no exposure to potential risk factors.[6–33]

It is clear that not all individuals exposed to MRSA will go on to develop full-blown infection. Many of them will be carriers of this organism and might contribute to its dissemination. Persons older than 65 years, women, individuals with diabetes and those who were in long-term care in the past year were more likely to have MRSA colonization.[34] However, most carriers actually do not have prior risk factors for this organism.[35] MRSA colonization occurs primarily in the nose, but other parts of the body such as axillae, perineal region or digestive tract can be affected.[36] The prevalence of MRSA colonization among different populations is difficult to establish because of many factors, such as sanitary practices. One U.S. study estimated the prevalence of MRSA carriers in hospitalized patients to be as high as 7%.[37]

## TYPES OF MRSA INFECTIONS

The spectrum of invasive MRSA infections is wide. Presentations, their diagnoses, prognoses and treatments are diverse. In this paper, important presentations are reviewed.

### Sepsis/endocarditis

MRSA bacteremia has increased recently because of invasive procedures, immune-compromised patients and growing resistance to antibiotic use. It has severe consequences in terms of morbidity and mortality because it can cause hematogenous spread to many organs.[38] Persistent bacteremia was independently associated with MRSA-infective endocarditis. This form of endocarditis was found to be more prevalent in the United States of America and Brazil than in other countries.[39] Right-sided MRSA endocarditis was also documented in intravenous drug users.[40]

### Respiratory tract

Lower respiratory tract infections with MRSA are frequently seen nowadays. It can occur with healthy individuals, but is common in persons with chronic respiratory conditions such as bronchiectasis, cystic fibrosis[41] and immune-compromised patients.[42] One of the dramatic presentations is necrotizing pneumonia. It is usually severe and can proceed rapidly to respiratory failure and death.[43] Patients with prolonged intubation or after undergoing tracheostomy are at an increased risk of developing MRSA respiratory infections, especially in intensive care and long-term facility settings.[44]

### Skin and soft tissue

Recent epidemiological trends have shown an increase in the rate of skin and soft tissue infections caused both by healthcare-associated and CA-MRSA.[45] These infections can be minor and self-limiting, such as furonculosis, to moderately severe, such as abscesses,[46] to the life-threatening Staphylococcal scalded skin syndrome.[47]

### Bone and joints

MRSA can disseminate hematogenously to bones causing acute osteomyelitis,[48] or joints and their surrounding structures, causing septic arthritis.[49] Articular and periarticular injections can also induce iatrogenic MRSA septic arthritis.[50] Surgical intervention, such as joint replacement procedures, can be complicated by serious challenging prosthetic joint MRSA infections. Treatment for such infections can be complicated and prolonged, requiring close collaboration of both surgical and medical teams.[51]

### Surgical sites and decubitus ulcers

Surgical wounds are a prime target for infection by MRSA, especially in hospitalized patients. In some surgical wards, this problem can become endemic and challenging to possible eradication.[52] Decubitus ulcers and possibly any chronic cutaneous ulcers can also be colonized and infected by MRSA. Patients in medical institutions and long-term care facilities are on top of the list for these types of infections.[53]

### Urinary tract

Although relatively rare, dissemination of MRSA to the urinary tract can occur via the blood stream or can ascend from the urethral meatus. Cases such as severe pyelonephritis were documented.[54] MRSA can colonize the urinary bladder and can occasionally cause urosepsis when conditions are opportune.[55]

### Other MRSA infections

MRSA can potentially infect any body system. In the hospital setting, nursing care, intensive antibiotic use and iatrogenic instrumentations promote topical and hematogenous dissemination of this resistant organism. However, more community-associated serious MRSA infections are being reported. Examples of this growing list of MRSA infections include meningitis,[56] epidural abscess,[57] neonatal liver abscess,[58] mastitis and toxic shock syndrome,[59] and even chorioamnionitis.[60]

## DIAGNOSIS

Diagnosing MRSA infection can vary from one presentation to another. It can be relatively easy to establish, such as in furuculosis, or might be difficult, like in subacute endocarditis. Clinically, fever and constitutional syptoms are frequently documented. Patients can experience a variety of other symptoms relating to the location of the infection. Some of these symptoms include productive cough, pleuritic chest pain, or pain at the site of an abcess, arthralgia/arthritis, loin pain or dysuria. Laboratory findings can guide toward establishing the correct diagnosis. In case of sepsis or deep purulent collection, leukocytosis with bandemia is frequently observed. However, leukopenia or normal white count might be present occasionally. Chemistry is ususally nonspecific. Acute renal injury can be secondary to dehydration or direct involvement of the urinary tract. In case of severe infection, acidosis is evidenced by a decrease in the bicarbonate and an elevation of the lactic acid levels. Coagulation profile can become abnormal if the infection leads to disseminated intravascular coagultation. Blood cultures are essential to confirm possible septicemia or endocarditis. Depending on the site of the infection, obtaining other body fluids, such as cerebrospinal or pleural fluids, purulent collection sample or devices, such as intravascular catheter, for culture and sensitivity are valuable for establishing the diagnosis. Recently, the U.S. Food and Drug Administration has approved a polymerase chain reaction assay that appears to be a valuable diagnostic test for quick differentiation of MRSA bacteremia.[61]

Computed tomography scanning, sonography (such as echocardiography) and endoscopic procedures are among some diagnostic tools used by clinicians to facilitate diagnosis of MRSA infections.

## TREATMENT

### Preventive measures

Nowadays, the battle lines with MRSA are drawn early. Strategies for infection control of this “superbug” are being developed. Many health care institutions and long-term facilities have adopted specific infection control programs aiming at controlling the spread of MRSA. These guidelines are complex and can be costly. Infection control has multiple important elements, such as early screening, identification of MRSA carries for isolation, nasal and skin decontamination, staff education, enforcement of hand hygiene and decontamination of patients' wards.[62] Some authors advocate stricter measures for the detection of MRSA. Besides nasal and cutaneous swabs, throat and rectal areas are considered for routine swabbing.[63] While these aggressive measures and strict guidelines can improve the efficacy of hospital bed usage,[64] they clearly cannot completely eradicate this resistant organism. It seems that MRSA is slowly gaining this battle by acquiring more territories and even using medical staff and their instruments, such as faucets, computer keyboards and stethoscopes, for further expansion.[65–66] Not only that, recent MRSA strains resistant to mupirocin, the topical antibiotic used for its eradication, are emerging.[67] Finally, investigators are working on an experimental vaccine to protect against MRSA. Results performed on mice are promising.[68]

### Antibiotics of choice for MRSA

Currently available effective antibiotics against MRSA are limited. For decades, intravenous vancomycin was the lone option for practicing physicians. It is notorious for its possible kidney damage, requiring strict serum concentration monitoring. Vancomycin can be administered intermittently or in a continuous fashion. The latter mode is probably more effective; however, no difference was observed between the two modalities regarding mortality and nephrotoxicity.[69] Recently, rare cases of VRSA started to emerge.[70] Newer antiobiotic choices include linezolid. It is the first available oxazolidinone antibacterial agent. It can be used both orally and through intravenous route. It has good activity against MRSA and offers a good alternative option to vancomycin in patients who have impaired renal function or with poor intravenous access. Linezolid is generally well tolerated, but can rarely have severe side-effects, such as thrombocytopenia and myelosuppression.[71] Daptomycin, a cyclic lipopeptide antibacterial agent derived from the fermentation of *Streptomyces roseosporus*, used intravenously, is also considered to be well tolerated and effective in the treatment of MRSA infections.[72] Tigecycline is less effective than the previous antibiotics. It still offers another choice in the treatment of MRSA infections, especially cutaneous ones.[73] Other antibiotics such as new glycopeptides (dalbavancin, oritavancin and telavancin), new anti-MRSA beta lactams (ceftobiprole) and new diaminopyrimidines (iclaprim) are currently in the pipeline for possible future use against MSRA.[74] Granulocyte colony-stimulating factor was tried experimentally and had no additional effect on survival and bacterial eradication in MRSA sepsis.[75]

### Other treatments

Depending on their presentations, MRSA infections can be simple or complicated. Occasionally, they can inflict severe organ damage, requiring further medical attention and therapeutic intervention. Examples include surgical drainage of a purulent collection. It can be relatively simple in the cutaneous form or may demand a major surgical intervention in case of deep abscesses. Severe forms of pneumonia can cause respiratory failure, requiring intubation, mechanical ventilation and all their possible iatrogenic complications. Chest tube insertion is required for empyema. Heart valvular replacement is carried out occasionally for valvular damage caused by endocarditis and removal of an infected prosthetic device is a main therapeutic intervention in MRSA orthopedic cases.

#### Contribution to MRSA drug resistance

The pattern of *S. aureus* resistance has been an evolving and challenging medical problem. It was initiated by first using Penicillin in the 1940s [Figure 1]. The emergence of MRSA in the early 1960s was a pivotal point in the evolution of this resistance. Indeed, despite efforts to stem its incidence, MRSA kept on gaining further ground inside and outside medical institutions. Not only that, physicians are starting to see resistance to vancomycin, which may be a clear indication that MRSA will keep developing resistance to any possible future antibiotic developed by scientists. Many contributing factors to MRSA resistance can be identified:

Antibiotic prescriptions' abuse by physicians: doctors have an ethical duty to provide their patients with the best possible care. Nowadays, most of them are practicing in an evolving health care environment with increasing rules and regulations that might interfere with their ethical and moral duties toward their patients. Indeed, it is safe to say that some physicians do not practice judicious antibiotic prescriptions because of either legal and/or financial possible repercussions.

Aggressive marketing by pharmaceutical companies: it is understandable that companies that invest billions of U.S. dollars in the development of medications should claim financial reward in case of successful discovery. However, there is a general trend that “big pharma” try to strike gold with their medications. They develop aggressive strategies, using huge personnel and huge amounts of money, to influence the perception of health care professionals and the public in general about their products. Their ultimate goal is to generate more sales. It is interesting to note that some patients can ask their doctors to provide them with a specific antibiotic by name! In some instances, it is difficult for doctors to convince them otherwise.

Patients' practices/sanitary conditions: patients' education about compliance in their care is extremely important. Nonadherence to an antibiotic regimen can exacerbate drug resistance. Some unfortunate individuals can be exposed to unsanitary conditions that facilitate the spread of MRSA. There is a collective duty by patients, health care professionals, legislators and other potential personnel to improve living conditions and access to better care.

Drug resistance cost: besides an estimated yearly heavy toll of around 100,000 deaths in the United States, health care-associated infections such as MRSA infections carry with them an exorbitant cost of $20 billion.[76] This gross estimation, even this high, remains relative. Indeed, some elements of this cost, such as hospitalization fees or drug cost, can be determined clearly; others, such of loss of productivity, are less than obvious. It has been proven, however, that MRSA eradication can contribute to better cost-effectiveness and alleviate the huge financial burden inflicted by this pathogen to the whole society.[77]

Finally, it must be clarified that establishing etiologies of MRSA resistance and writing guidelines for infection control can be a relatively easy task. However, implementing ways to reduce it can face many barriers, such as poor health education, limited financial resources, poor infrastructures, undertrained staff, inadequate laboratories and shortage in isolation rooms and beds.[78]

## CONCLUSIONS

MRSA is a virulent pathogen that appeared more than four decades ago. It is spreading worldwide within hospitals, extended care facilities and the community at large. It is evolving into a growing epidemic, increasingly claiming victims. Despite sophisticated strategies and costly efforts to limit the growth of this epidemic, the overall results are less than ideal. It is clear that further collaborative educated measures and work, by health care professionals and pharmaceutical companies and legislators, are needed to stem the severe consequences of this “superbug.”
